# Leveraging digital pathology to enhance breast cancer diagnosis in low-resource settings: a cross-sectional study of major histopathology laboratories in Uganda

**DOI:** 10.1093/oodh/oqag034

**Published:** 2026-09-03

**Authors:** Agnes R Semwanga, James Serubugo, Vincent M Kiberu, Hawa Nalwoga, Evelyn K Kahiigi, Nazarius M Tumwesigye, Sam Kalungi

**Affiliations:** Department of Information Systems, Makerere University College of Computing and Information Sciences, University Road P.O. Box 7062, Kampala, Uganda; Department of Epidemiology and Biostatistics, Makerere University School of Public Health, New Mulago Hill Road, Mulago P.O. Box 7072, Kampala, Uganda; Department of Pathology, School of Biomedical Sciences, Makerere University College of Health Sciences, Pathology-Microbiology Building Upper Mulago Hill Road, Mulago P.O. Box 7072, Kampala, Uganda; Department of Epidemiology and Biostatistics, Makerere University School of Public Health, New Mulago Hill Road, Mulago P.O. Box 7072, Kampala, Uganda; Department of Pathology, School of Biomedical Sciences, Makerere University College of Health Sciences, Pathology-Microbiology Building Upper Mulago Hill Road, Mulago P.O. Box 7072, Kampala, Uganda; Department of Information Systems, Makerere University College of Computing and Information Sciences, University Road P.O. Box 7062, Kampala, Uganda; Department of Epidemiology and Biostatistics, Makerere University School of Public Health, New Mulago Hill Road, Mulago P.O. Box 7072, Kampala, Uganda; Uganda National Cancer Institute, Upper Mulago Hill Road P.O. Box 3935, Kampala, Uganda

**Keywords:** digital pathology, web-based system, breast cancer, observer agreement

## Abstract

Breast cancer remains a leading cause of morbidity and mortality among Ugandan women, particularly in rural areas where pathology infrastructure and specialist access are limited. This study aimed to improve diagnostic efficiency and accuracy by digitizing breast cancer slides and implementing a web-based histopathology system to support remote diagnostics. A total of 100 glass slides from core breast biopsy samples were digitized using an Aperio AT2 slide scanner. A web-based breast histology information management system (BHIMS) was developed to store and provide remote access to these digital slides. Two experienced pathologists independently evaluated the slides using both conventional microscopy and the digital platform. Assessments focused on key morphological features, including cancer diagnosis, type, grade, mitosis, lymphatic invasion, nuclear pleomorphism, and tubule formation. Inter- and intra-observer agreement was analyzed using kappa statistics. Digital pathology showed high inter- and intra-observer reliability, particularly for cancer diagnosis and type. Slightly lower agreement was noted for nuanced features such as mitosis and lymphatic invasion. Conventional microscopy yielded stronger observer agreement and confidence, especially for mitotic count and invasion. Digital pathology offers a promising approach to improve breast cancer diagnosis in resource-limited settings. The BHIMS system enabled remote access and collaborative review of cases, supporting decentralized pathology services. However, broader adoption will require investment in high-resolution scanners, reliable internet, data storage, and training of pathologists. With these enhancements, digital pathology could significantly strengthen cancer care delivery in rural Uganda.

## German version (DEUTSCH)

**Hintergrund:**

Brustkrebs ist weiterhin eine der führenden Ursachen für Morbidität und Mortalität bei Frauen in Uganda, insbesondere in ländlichen Gebieten, wo die pathologische Infrastruktur und der Zugang zu Fachärzten begrenzt sind. Ziel dieser Studie war es, die diagnostische Effizienz und Genauigkeit durch die Digitalisierung von Brustkrebspräparaten und die Implementierung eines webbasierten histopathologischen Systems zur Unterstützung der Ferndiagnostik zu verbessern.

**Methoden:**

Insgesamt wurden 100 Glasobjektträger aus Stanzbiopsien der Brust mithilfe eines Aperio AT2-Scanners digitalisiert. Ein webbasiertes Breast Histology Information Management System (BHIMS) wurde entwickelt, um diese digitalen Präparate zu speichern und den Fernzugriff zu ermöglichen. Zwei erfahrene Pathologen bewerteten die Präparate unabhängig voneinander sowohl mittels konventioneller Mikroskopie als auch über die digitale Plattform. Die Bewertung konzentrierte sich auf wesentliche morphologische Merkmale, darunter Krebsdiagnose, Typ, Grad, Mitoseaktivität, lymphatische Invasion, nukleäre Pleomorphie und Tubulusbildung. Die Inter- und Intra-Beobachter-Übereinstimmung wurde mithilfe der Kappa-Statistik analysiert.

**Ergebnisse:**

Die digitale Pathologie zeigte eine hohe Inter- und Intra-Beobachter-Zuverlässigkeit, insbesondere bei der Krebsdiagnose und Klassifikation. Eine etwas geringere Übereinstimmung wurde bei differenzierteren Merkmalen wie der Mitoseaktivität und der lymphatischen Invasion festgestellt. Die konventionelle Mikroskopie wies eine stärkere Beobachterübereinstimmung und ein höheres Vertrauen auf, insbesondere bei der Bewertung der Mitosezahl und der Invasion.

**Schlussfolgerung:**

Die digitale Pathologie stellt einen vielversprechenden Ansatz zur Verbesserung der Brustkrebsdiagnostik in ressourcenbegrenzten Umgebungen dar. Das BHIMS-System ermöglichte den Fernzugriff und die kollaborative Fallbewertung und unterstützte damit dezentrale pathologische Dienste. Für eine breitere Anwendung sind jedoch Investitionen in hochauflösende Scanner, zuverlässige Internetverbindungen, Datenspeicherinfrastruktur sowie die Schulung von Pathologen erforderlich. Mit diesen Verbesserungen könnte die digitale Pathologie die Krebsversorgung in ländlichen Gebieten Ugandas erheblich stärken.

**Schlüsselwörter:**

digitale Pathologie, webbasiertes System, Brustkrebs, Beobachterübereinstimmung

## Version Française

**Contexte:**

Le cancer du sein demeure une cause majeure de morbidité et de mortalité chez les femmes en Ouganda, en particulier dans les zones rurales où les infrastructures en anatomopathologie et l’accès aux spécialistes sont limités. Cette étude visait à améliorer l’efficacité et la précision diagnostiques en numérisant des lames de cancer du sein et en mettant en place un système histopathologique basé sur le web pour soutenir le diagnostic à distance.

**Méthodes:**

Un total de 100 lames issues de biopsies mammaires à l’aiguille ont été numérisées à l’aide d’un scanner de lames Aperio AT2. Un système de gestion de l’information en histologie mammaire basé sur le web (BHIMS) a été développé afin de stocker ces lames numériques et de permettre un accès à distance. Deux pathologistes expérimentés ont évalué indépendamment les lames à la fois par microscopie conventionnelle et via la plateforme numérique. L’évaluation portait sur des caractéristiques morphologiques clés, notamment le diagnostic du cancer, le type, le grade, les mitoses, l’invasion lymphatique, le pléomorphisme nucléaire et la formation de tubules. L’accord inter- et intra-observateur a été analysé à l’aide de la statistique kappa.

**Résultats:**

La pathologie numérique a montré une forte fiabilité inter- et intra-observateur, en particulier pour le diagnostic du cancer et sa classification. Un accord légèrement plus faible a été observé pour des caractéristiques plus subtiles telles que l’activité mitotique et l’invasion lymphatique. La microscopie conventionnelle a présenté un accord entre observateurs plus élevé ainsi qu’un niveau de confiance supérieur, notamment pour l’évaluation du nombre de mitoses et de l’invasion.

**Conclusion:**

La pathologie numérique constitue une approche prometteuse pour améliorer le diagnostic du cancer du sein dans les contextes à ressources limitées. Le système BHIMS a permis un accès à distance et une évaluation collaborative des cas, soutenant ainsi la décentralisation des services de pathologie. Toutefois, une adoption à grande échelle nécessitera des investissements dans des scanners haute résolution, une connectivité Internet fiable, des infrastructures de stockage de données ainsi que la formation des pathologistes. Avec ces améliorations, la pathologie numérique pourrait renforcer considérablement la prise en charge du cancer dans les zones rurales en Ouganda.

**Mots-clés:**

pathologie numérique, système basé sur le web, cancer du sein, accord inter-observateur

## Introduction

Breast cancer remains a major global health concern, ranking as the most common cancer among women and the leading cause of cancer-related deaths in many parts of the world (Wilkinson and Gathani 2022). Early detection significantly improves prognosis and reduces treatment costs, yet in low-resource settings like Uganda, timely diagnosis remains a major challenge. In Uganda, breast cancer is the second most common cancer after cervical cancer, with age-standardized incidence and mortality rates of 21.3 and 10.3 per 100 000 women, respectively (Ekdahl Hjelm et al. 2019). The 5-year survival rate for breast cancer in Uganda is between 46% and 56%, significantly lower than rates above 80% seen in high-income countries (Joko-Fru et al. 2020). This disparity is largely due to late-stage presentation, with up to 89% of Ugandan women diagnosed at Stage III or IV when treatment is less effective (Galukande et al. 2015).

Timely diagnosis is hindered by limited pathology infrastructure, a severe shortage of trained pathologists, and logistical challenges in transporting specimens to centralized laboratories, primarily Mulago National Referral Hospital (Ruhangaza et al. 2024). Manual diagnostic workflows, including glass slide microscopy and histological staining, dominate the current system. These traditional methods, while foundational to cancer diagnosis (Kayser et al. 2011), are constrained by their fragility, degradation over time, and limited accessibility for collaborative or remote consultations (Zia et al. 2025).

Technological advancements in medical imaging and informatics offer promising alternatives. Radiographic imaging techniques—such as mammography, computed tomography, magnetic resonance imaging, and histopathological imaging—have enhanced early-stage breast cancer detection (Kamali et al. 2022). Emerging computational techniques, including machine learning and deep learning, have demonstrated remarkable potential in image-based diagnostics by improving classification accuracy and handling large volumes of data (Kamali et al. 2022; Madani et al. 2022). Tools like the Weka data mining platform, employing dimensionality reduction methods like WrapperSubsetEval, have improved model performance in breast cancer prediction (Harmouch 2015; Agrawal 2019).

Digital pathology, the conversion of glass slides into high-resolution digital images, is increasingly adopted in high-income settings to support telepathology, collaborative diagnostics, and artificial intelligence (AI) integration (Fatima et al. 2024). Digital slides are durable, easy to annotate, and can be transmitted instantly for review, supporting improved diagnostic turnaround, especially in remote or underserved areas. As highlighted by Philip et al. (2024), combining manual, semi-automatic, and automatic imaging approaches enhances diagnostic reliability. Biomedical imaging informatics enables the acquisition, representation, and sharing of diagnostic images in formats interpretable by health professionals, thereby extending the capabilities of resource-constrained systems.

In Uganda, the widespread adoption of digital pathology remains limited, especially in rural hospitals that often lack histopathology laboratories and the human resource capacity for timely diagnosis. Delays caused by the manual transportation of glass slides can stretch into months, diminishing the effectiveness of treatment and contributing to preventable mortality (Kiwanuka et al. 2020; Betmouni 2021). Moreover, glass slides are not amenable to real-time sharing or centralized diagnosis, further limiting access to timely expert review (Pantanowitz et al. 2011; Jahn et al. 2020).

This study therefore aimed to address these gaps by digitizing histopathology slides for breast cancer specimens, analyzing the workflow and infrastructure challenges affecting breast cancer diagnosis in rural Uganda, and developing a digital histopathology reporting system. The study also evaluated diagnostic agreement between observers using digital versus conventional glass slides to assess the feasibility of digital pathology for routine diagnostic use. The findings aim to inform efforts to decentralize cancer diagnostics and improve outcomes through digital transformation of histopathology services in low-resource settings.

## Materials and methods

### Study design

This study employed a mixed-method, cross-sectional design implemented in three sequential phases: (Wilkinson and Gathani 2022) digitization of histopathology glass slides (Ekdahl Hjelm et al. 2019), development and deployment of a web-based digital histopathology reporting system (Joko-Fru et al. 2020), and morphological comparison of diagnoses made from both glass and digital breast cancer slides. The study aimed to assess the reliability and usability of digitized histopathology slides in breast cancer diagnosis in Uganda.

### Study setting

The study was conducted across four major histopathology laboratories in Uganda: The Department of Pathology at Mbarara University of Science and Technology, Uganda Cancer Institute, the Department of Pathology at Makerere University, and Kampala International University Teaching and Research Hospital (Western Campus). St. Mary’s Hospital Lacor was also assessed but did not meet the final inclusion criteria. Glass slides included in the study were prepared from formalin-fixed paraffin-embedded (FFPE) tissue blocks of patients with histologically confirmed breast cancer between January 2020 and March 2021. Patient identification information was removed to ensure confidentiality and only slide identification numbers were used for reference. Institutional Review Board (IRB) approvals and official permissions were obtained from each participating institution.

### Sampling and sample size determination

The sample size was determined using the Kish and Leslie formula (Wiegand 1968) for single population proportion:


$${N}_s=\frac{Z_a^2\cdot P\left(1-P\right)}{S^2}$$

where

*N_s_* = required sample size (number of slides),

*Z_a_* = standard normal value for 95% confidence level = 1.96,

*P* = estimated prevalence of histologically diagnosed breast cancer = 0.25,

*S* = margin of error = 0.05.

This yielded a total of 100 breast cancer slide cases. A purposive sampling technique was used to recruit two experienced pathologists (minimum of 2 years of experience in breast cancer diagnosis) to read the slides. The study included two pathologists due to the limited availability of qualified specialists in breast cancer diagnosis within the study settings. Both observers met the minimum experience criteria, and their inclusion was sufficient to support inter- and intra-observer agreement analysis, consistent with similar diagnostic reliability studies in resource-limited settings.

Cases of male breast cancer and diagnoses made outside the study timeframe were excluded.

### Slide digitization process

Glass slides were prepared from FFPE tissue blocks and digitized using a high-resolution slide scanner. Digital images were captured using a Canon EOS 90D digital camera (Canon Inc., Tokyo, Japan) with a resolution of 32.5 megapixels, in RAW format, under standardized lighting conditions. The device was calibrated regularly, and inherent limitations such as sensor noise, dynamic range, and color accuracy were considered during image analysis. The digitization process consisted of four main steps: image acquisition (scanning), image storage, image editing, and image display. The resulting digital slides were uploaded to the newly developed digital histopathology reporting system for validation and diagnostic assessment.

### Development of the digital histopathology reporting system

The system was developed using the traditional System Development Life Cycle methodology. Tool refinement/development involved collecting/gathering input from pathologists, laboratory technologists, and managers to define the system’s features and functional requirements. The system was designed using Microsoft Visio, developed using object-oriented PHP (PHP: Hypertext Preprocessor), and supported by a MySQL database. Upon completion, the system was subjected to user testing and validation workshops with key stakeholders, including pathologists, laboratory staff, and health policymakers.

The breast histology information management system (BHIMS) platform was developed as a prototype for research and validation purposes and is not currently open source. Access is restricted to the study team and participating institutions, with plans to explore broader access to support scalability and adoption.

### Validation and observer agreement assessment

The diagnostic validity of digital slides was assessed using both intra- and inter-observer agreement tests. Seven diagnostic variables were evaluated: diagnosis similarity, morphological features, nuclear pleomorphism, mitoses, cancer grade, lymphatic invasion, and interpretation time.

### Slide evaluation and usability assessment

The evaluation process began with both pathologists independently reviewing the original glass slides using conventional light microscopy. Each morphological feature was assessed using binary scores (“yes” = 1, “no” = 0), except for cancer grade, which was scored on a three-point ordinal scale (Ekdahl Hjelm et al. 2019; Joko-Fru et al. 2020; Wilkinson and Gathani 2022). Following a 2-week washout period to minimize recall bias, the same pathologists assessed the digitized slides—assigned blinded alphanumeric codes—using identical evaluation criteria. Each slide was uniquely identified (slides 1–100 for glass; alpha-coded for digital), and the maximum possible score per slide was 9, achieved when all features were positive and the cancer grade was marked as 3. Intra-observer agreement was calculated by comparing each pathologist’s scores across the two modalities (glass and digital), while inter-observer agreement assessed using [Cohen’s kappa/Fleiss’ kappa/intraclass correlation coefficient], with two independent observers evaluating all images to ensure consistency and reliability of the measurements.

To evaluate the usability of digital slides, both pathologists assessed four key aspects: (Wilkinson and Gathani 2022) the ability of digital slides to support diagnoses comparable to those made using glass slides (Ekdahl Hjelm et al. 2019), the ability to observe similar morphological features (Joko-Fru et al. 2020), the completeness with which diagnostic features were captured (Galukande et al. 2015), and the similarity in time required for interpretation. Responses to each usability criterion were recorded using a binary format (“yes” or “no”).

### Statistical analysis

Quantitative data were collected in Microsoft Excel and analyzed using Stata version 13. The Kappa coefficient was used to assess both intra- and inter-observer agreement levels, with values closer to 1 indicating stronger agreement. Additionally, *t*-tests and descriptive cross-tabulations were used to analyze observer ratings and evaluate the acceptability of digital slides in breast cancer diagnosis.

### Study workflow diagram

**Figure 1:**
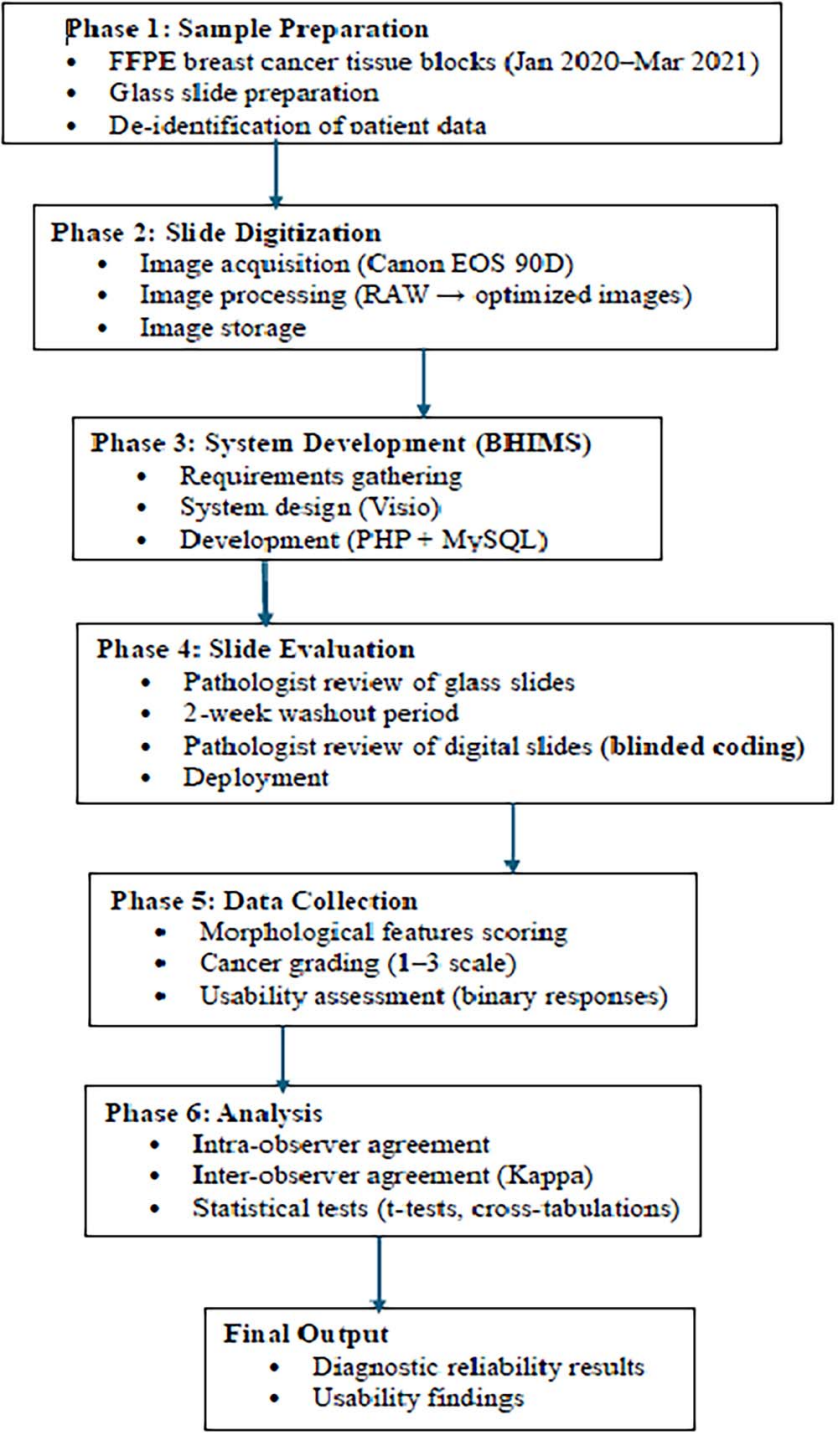
Showing workflow diagram for this study.

A schematic workflow diagram summarizing the study design and procedures has been included (Fig. 1). The diagram illustrates the sequential phases of the study, including slide selection, digitization, system development, diagnostic evaluation, and statistical analysis.

## Results

### Slide characteristics and participants

A total of 100 digitized breast cancer histopathology slides were successfully created from FFPE blocks. The slides represented cases diagnosed between January 2020 and March 2021. Two qualified pathologists, each with more than 2 years of experience in breast cancer diagnosis, participated in the diagnostic evaluation. All participating laboratories (*n* = 4) contributed validated glass slides for digitization and comparison.

### Breast histology information management system

**Figure 2:**
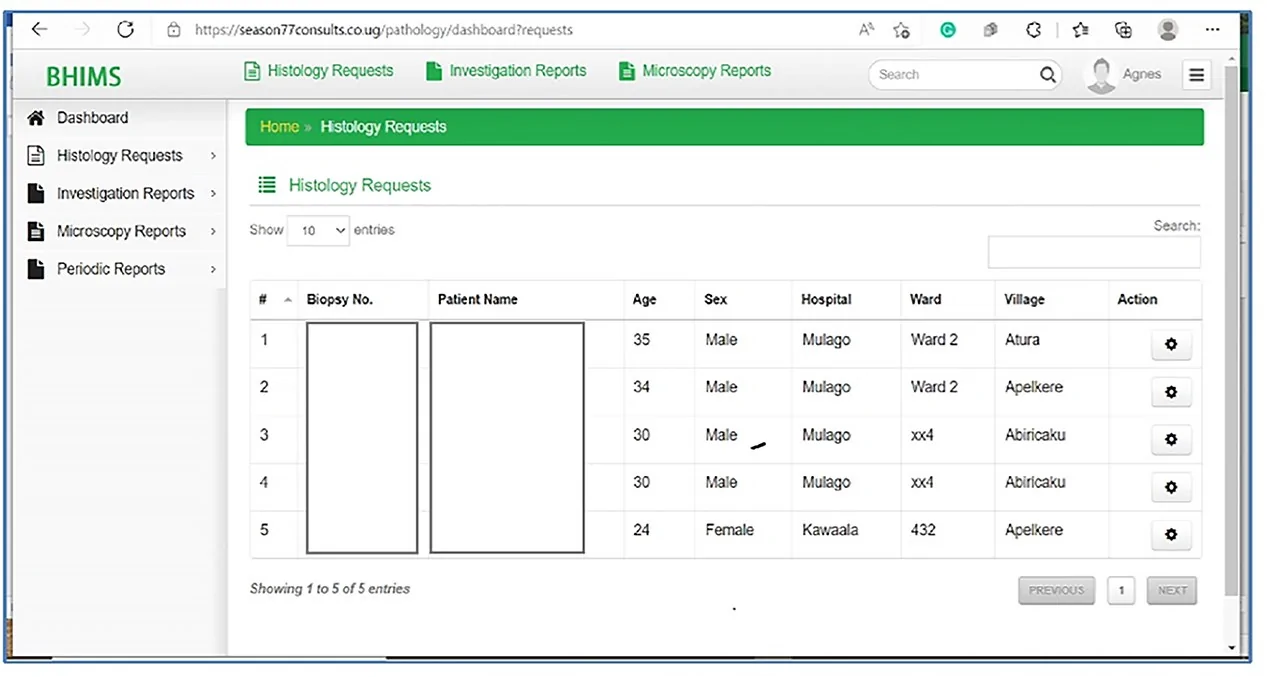
Showing a dashboard of the screenshots of BHIMS.

BHIMS, a web-based histopathology repository system, was developed to support the business processes for breast cancer detection, diagnosis, and early treatment, especially in rural areas of Uganda where medical infrastructure and expertise for breast cancer patients are limited or nonexistent. BHIMS (dashboard screenshot shown in Fig. 2) accurately captures and stores slides in digital format for the assessment of morphological characteristics of core breast cancer. The system maintains electronic reports and prognosis information to support real-time patient care and management, offering the following functionalities:

a) E-histopathology reporting: Conventional microscopy is performed and viewed using electronic devices such as computer screens, laptops, or other devices at the pathologist’s convenience. This enables telepathology and decentralization of pathological technical services, allowing histology slides to be sent to a centralized histopathology center for reporting.
b) E-histopathology consultations: Slides can be shared over a network with multiple histopathology consultants without requiring physical transportation. This facilitates consultative reviews for obtaining second opinions on histopathology diagnoses and makes teleconferencing among pathologists possible for case diagnosis.
c) E-tumor board sessions: The system provides pathologists with an efficient way to present their slides to various teams of oncologists and physicians at a distance while discussing patient management based on diagnosis, tumor type, prognosis, and effective treatment methods.

### Intra-observer agreement between glass and digital slides

The intra-observer agreement for diagnoses between glass and digital slides across seven diagnostic variables demonstrated substantial to almost perfect agreement based on Cohen’s Kappa statistics. The results are summarized in Table 1.

**Table 1:** Intra-observer agreement on diagnostic variables (*N* = 100).

Diagnostic variable	Kappa (*κ*)	Interpretation
Overall diagnosis	0.86	Almost perfect
Morphological features	0.81	Almost perfect
Nuclear pleomorphism	0.78	Substantial
Mitoses	0.74	Substantial
Cancer grade	0.72	Substantial
Lymphatic invasion	0.76	Substantial
Interpretation time	0.69	Moderate to substantial

These results indicate that digital slides closely mirror traditional glass slides in supporting accurate diagnoses and consistent interpretations by the same observer. Inter-Observer Agreement.

When comparing evaluations between the two pathologists across slide formats, inter-observer agreement remained substantial, though slightly lower than intra-observer agreement.

This suggests general consistency between observers when using either glass or digital formats.

### Inter-rating by the same methods using different raters

Kappa coefficients were calculated to assess agreement between observers across seven diagnostic aspects—cancer diagnosis, cancer type, nuclear pleomorphism, tubule formation, cell mitosis, cell invasion, and cancer grade—based on evaluations of 100 breast cancer specimens. Levels of agreement were interpreted as follows: moderate (*κ* = 0.41–0.60) and excellent (*κ* = 0.81–1.00), based on standard interpretation guidelines. A detailed classification table is provided in Tables 1 and 2. Results are summarized in Tables 1 and 2.

**Table 2:** Inter-observer agreement across slide formats (*N* = 100).

Diagnostic variable	Kappa (*κ*)	Interpretation
Overall diagnosis	0.78	Substantial
Morphological features	0.74	Substantial
Nuclear pleomorphism	0.70	Substantial
Mitoses	0.66	Moderate
Cancer grade	0.61	Moderate
Lymphatic invasion	0.69	Moderate to substantial
Interpretation time	0.65	Moderate

### Performance on digital and glass slides

When evaluating digital slides, Observer 1 achieved the highest positive agreement for cancer diagnosis (76%), followed by cancer type (68%), with the lowest agreement observed in cancer grade (55%). Similarly, Observer 2 recorded the highest positive agreement for cancer diagnosis (59%) and cancer type (55%), with the lowest scores for cancer invasion (35%) and cancer grade (39%). These findings highlight a consistent trend where diagnostic features such as cancer diagnosis and type were more reliably identified on digital slides compared to more nuanced histological features like invasion and grading.

On glass slides, Observer 1 recorded the highest positive results for cancer type (69%) and cancer diagnosis (68%). Observer 2 demonstrated the strongest performance on nuclear pleomorphism (62%), followed closely by cancer diagnosis and cancer type, each at 61%. However, both observers showed reduced agreement for cell invasion, which had the lowest positive results—39% for Observer 1 and just 27% for Observer 2—indicating greater difficulty in identifying this feature using traditional microscopy.

### Inter-observer agreement

Kappa coefficients for the different aspects (cancer diagnosis, cancer type, nuclear pleomorphism, tubule formation, cell mitosis, cell invasion, and cancer grade) of the 100 specimens were obtained as shown in Tables 3 and 4. The distribution of participant characteristics and key outcome measures are summarized in Table 3, while detailed subgroup analyses and comparative results are presented in Table 4.

**Table 3:** Showing percentages of positives, negative and agreements for various parameters using same methods (digital-digital and glass–glass) but different raters, N = 100.

		Diagnosis			Cancer type			Nuclear Pleomorphism			Tubule Formation			Mitosis seen			Invasion seen			Cancer Grade		
Method	Rater	+	-	A	+	-	A	+	-	A	+	-	A	+	-	A	+	-	A	+	-	A
		%	%	%	%	%	%	%	%	%	%	%	%	%	%	%	%	%	%	%	%	%
Digital	1	59	41	85	58	41	87.9	57	43	85.0	60	40	82.0	54	46	76.0	55	45	72.0	55	45	76.0
	2	52	48		54	45		50	50		52	48		54	46		35	65		39	61	
Glass	1	68	32	81.0	69	31	80.0	61	39	83.0	56	44	83.0	48	52	54.0	39	61	86.0	55	45	84.0
	2	61	39		61	39		62	38		59	41		52	48		27	73		39	61	

*Note: + means slide with Positive result, − means slide with negative result and A-Means agreement on result*

**Table 4:** Showing kappa statistics for using same methods (digital-digital and glass–glass) but different raters, *N* = 100.

		Diagnosis		Cancer type		Nuclearpleomorphism		Tubule formation		Mitosis seen		Invasion seen		Cancer Grade	
Method	Rater	*k*	*_P*	*K*	*_P*	*K*	*_P*	*K*	*_P*	*k*	*_P*	*K*	*_P*	*K*	*_P*
Digital	1 and 2	0.70	***	0.754	***	0.700	***	0.637	***	0.517	***	0.456	***	0.530	***
Glass	1 and 2	0.587	***	0.564	***	0.641	***	0.652	***	0.082	0.207	0.689	***	0.687	***

*Note*: *k*, kappa correlation coefficient; *P, P*-value; ^*^means = <.05; ^**^means = <.01; ^***^means = <.001.

For digital slides, the highest inter-observer agreement was recorded for cancer type, with 87.9% agreement (*κ* = 0.754, *P* < .001), suggesting substantial reliability. Cancer diagnosis and nuclear pleomorphism followed closely, each with 85% agreement (*κ* = 0.700, *P* < .001). Tubule formation also showed strong consistency, with 82% agreement (*κ* = 0.640, *P* < .001). The lowest inter-observer agreement was found in cell invasion, with 72% agreement (*κ* = 0.460, *P* < .001), reflecting moderate reliability. Digital inter-observer agreement was highest for cell mitosis and cancer grade (76% each); however, cell invasion (72%) was highlighted as the representative measure because it reflects the most clinically relevant functional feature of tumor behavior, and its reproducibility is critical for subsequent analyses. Overall, digital slides demonstrated substantial inter-observer agreement in four out of the seven diagnostic categories and moderate agreement in the remaining three.

In comparison, glass slide evaluations showed the highest inter-observer agreement for cell invasion (86%, *κ* = 0.690, *P* < .001) and cancer grade (84%, *κ* = 0.690, *P* < .001), both indicating substantial reliability. However, cell mitosis had the lowest agreement between observers, with only 54% agreement (*κ* = 0.080, *P* > .05), suggesting poor inter-rater reliability for this feature when assessed using conventional microscopy.

Overall, digital slides provided a more consistent level of diagnostic reliability across most parameters. These findings support the adoption of digital pathology systems like BHIMS in resource-limited settings to enhance diagnostic accuracy and facilitate specialist consultations.

### Intra-observer agreement

For intra-observer agreement, each rater applied both methods sequentially. Tables 5 and 6 show the results where specimens were analyzed using glass slides, followed by digital slides.

**Table 5:** Showing percentages of positives, negative and agreements for various parameters using same raters but different methods, N = 100.

		Diagnosis			Cancer type			Nuclear Pleomorphism			Tubule Formation			Mitosis seen			Invasion seen			Cancer Grade		
Method	Rater	+	-	A	+	-	A	+	-	A	+	-	A	+	-	A	+	-	A	+	-	A
		%	%	%	%	%	%	%	%	%	%	%	%	%	%	%	%	%	%	%	%	%
Digital	1	59	41	85	58	41	87.9	57	43	85.0	60	40	82.0	54	46	76.0	55	45	72.0	55	45	76.0
	2	52	48		54	45		50	50		52	48		54	46		35	65		39	61	
Glass	1	68	32	81.0	69	31	80.0	61	39	83.0	56	44	83.0	48	52	54.0	39	61	86.0	55	45	84.0
	2	61	39		61	39		62	38		59	41		52	48		27	73		39	61	

*Note*: + means slide with Positive result, − means slide with negative result and A—Means agreement on result.

**Table 6:** Kappa coefficients (k) for using same raters but different methods, N = 100.

		Diagnosis		Cancer type		Nuclearpleomorphism		Tubule formation		Mitosis seen		Invasion seen		Cancer Grade	
Raters		k	_p	k	_p	k	_p	k	_p	k	_p	k	_p	k	_p
1	Glass & Digital	0.914	***	9.724	***	0.686	***	0.589	***	0.522	***	0.376	***	0.396	***
2	Glass & Digital	0.856	***	0.554	***	0.543	***	0.247	**	0.517	0.058	0.072	0.232	0.201	*

*Note*: k, kappa correlation coefficient; *P, P*-value, ^*^ means = < .05; ^**^means = < .01; ^***^means = < .001.

Intra-observer agreement was evaluated by having each pathologist (Observer 1 and Observer 2) assess the same set of 100 breast cancer specimens using both glass and digital slides, with a 2-week washout period between the two assessments. This approach enabled comparison of each observer’s consistency in diagnostic interpretation across the two modalities. The diagnostic performance of digital slides was comparable to that of glass slides and did not differ significantly between the two modalities. The results are summarized below.

Observer 1 demonstrated the highest positive agreement in cancer diagnosis, with 79% agreement on glass slides and 76% on digital slides. Cancer type followed closely, showing consistent agreement at 68% across both modalities. The lowest positive agreement was observed for lymphatic invasion (41%). Statistical analysis revealed excellent intra-observer reliability for cancer diagnosis (agreement = 97%, *κ* = 0.914, *P* < .001) and substantial reliability for cancer type (agreement = 88%, *κ* = 0.724, *P* < .001). However, only fair agreement was noted for lymphatic invasion (*κ* = 0.376, *P* > .001).

Observer 2 recorded the highest positive results for tubule formation (64%), followed by cancer diagnosis (59%) and cancer type (58%). The lowest positive results were found for cell invasion (27%) and cancer grade (39%). In terms of intra-observer reliability, cancer diagnosis showed excellent agreement (agreement = 93%, *κ* = 0.700, *P* < .001). Moderate agreement was observed for cancer type (*κ* = 0.554, *P* < .001), nuclear pleomorphism (*κ* = 0.543, *P* < .001), and mitoses (*κ* = 0.517, *P* < .001). The lowest reliability scores were recorded for lymphatic invasion (*κ* = 0.072, *P* < .005), indicating only slight agreement, and for cancer grade (*κ* = 0.201, *P* < .005), indicating fair agreement. In summary, Observer 2 demonstrated moderate to excellent intra-observer agreement in four of the seven diagnostic variables, with low reliability observed in assessing lymphatic invasion and cancer grade.

These findings suggest that both observers showed strong consistency in identifying key diagnostic features such as cancer diagnosis and type across glass and digital slides. However, diagnostic elements that rely on more nuanced morphological features, such as lymphatic invasion and grading, yielded lower intra-observer agreement, underscoring the complexity of these criteria and the potential need for enhanced digital imaging tools or additional training.

The intra-observer analysis comparing digital and glass slide assessments showed strong consistency in breast cancer diagnosis, particularly for key parameters such as cancer diagnosis and type. Observer 1 demonstrated excellent agreement for cancer diagnosis (*κ* = 0.914) and substantial agreement for cancer type (*κ* = 0.724), while Observer 2 showed similarly high consistency for cancer diagnosis (*κ* = 0.70). Moderate agreement was noted in both observers for features like nuclear pleomorphism and mitotic count.

However, both observers recorded the lowest agreement on lymphatic invasion (Observer 1: *κ* = 0.376; Observer 2: *κ* = 0.072) and cancer grading, indicating variability in interpretation for these complex features. Observer 1 demonstrated good to excellent intra-observer agreement in three of the seven diagnostic parameters—cancer diagnosis, cancer type, and nuclear pleomorphism. Moderate agreement was observed for tubule formation and mitotic figures, while fair agreement was noted for lymphatic invasion and overall cancer grade.

Overall, Observer 1 achieved moderate to good agreement in five out of the seven parameters. In comparison, Observer 2 demonstrated moderate to excellent agreement in four of the seven parameters.

## Discussion

This study evaluated the diagnostic reliability of digital histopathology compared to conventional glass slide microscopy in the assessment of breast cancer cases in a low-resource setting, specifically Uganda. The findings demonstrated that digital slides provided diagnostic performance comparable to glass slides, with substantial intra- and inter-observer agreement across core histopathological parameters. These results reinforce the growing body of evidence that digital pathology is a viable and effective alternative to conventional microscopy, especially for core diagnostic features (Snead David et al. 2016; Emily et al. 2017; Azam et al. 2021). Importantly, these findings provide a strong foundation for the progressive scale-up of digital pathology systems within Uganda’s health system and similar low-resource contexts.

### Diagnostic reliability and observer agreement in the context of scale-up

High inter-observer agreement achieved using digital slides, particularly for cancer type (*κ* = 0.754) and cancer diagnosis (*κ* = 0.70), indicates that digital pathology can reliably support routine diagnostic workflows at scale. These findings are consistent with previous studies that reported high concordance between digital and glass slide diagnoses for primary cancer characteristics (Snead David et al. 2016; Sanjay et al. 2018). As such, digital platforms can be confidently integrated into national diagnostic frameworks, especially for high-burden cancers such as breast cancer.

However, slightly higher agreement on lymphatic invasion and cancer grade observed with glass slides highlights important considerations for scale-up. These differences may be attributed to limitations in digital image resolution and depth perception, which are critical for assessing nuanced histological features. Similar observations by (Emily et al. 2017) suggest that certain diagnostic tasks—such as identifying mitotic figures and subtle nuclear details—may require enhanced imaging technologies or hybrid diagnostic approaches during early phases of implementation.

Intra-observer agreement further supports the feasibility of scaling digital pathology. Observer 1 demonstrated excellent agreement in cancer diagnosis (*κ* = 0.914), while Observer 2 also showed high reliability (*κ* = 0.856), confirming that trained pathologists can consistently interpret digital slides. This underscores the importance of capacity building and standardized training programs as part of scale-up strategies, ensuring that pathologists develop proficiency in digital slide interpretation.

Nevertheless, lower agreement for nuanced features such as mitoses and lymphatic invasion in both intra- and inter-observer assessments signals critical areas requiring targeted intervention. The notably low inter-observer agreement for mitotic count on glass slides (*κ* = 0.082) and only fair reliability for invasion on digital slides (*κ* = 0.376) are consistent with findings from Williams et al. (2018) and Meyer et al. (2005). For scale-up, these gaps highlight the need for enhanced quality assurance protocols, advanced imaging capabilities, and continuous professional development, as well as potential integration of computer-aided diagnostic tools to improve consistency in interpreting subtle morphological features.

### Utility and impact of BHIMS: Implications for national scale-up

The implementation of the BHIMS represents a critical step toward the digitization and decentralization of pathology services in Uganda. By enabling remote access to digital slides, BHIMS supports timely and collaborative diagnosis across geographically dispersed locations. This is particularly valuable in rural and underserved regions where pathologist shortages and logistical challenges in transporting physical slides remain significant barriers (Williams et al. 2018; Hanna et al. 2019).

For national scale-up, BHIMS demonstrates the feasibility of establishing a hub-and-spoke model, where central reference laboratories support peripheral health facilities through telepathology. The system’s ability to facilitate e-histopathology consultations and virtual tumor board discussions further strengthens multidisciplinary care, improving diagnostic accuracy, treatment planning, and patient outcomes.

### Next steps for scale-up and sustainability

To translate these findings into sustainable health system improvements, several key steps are recommended:

1) Infrastructure Strengthening: Investment in high-resolution slide scanners, reliable internet connectivity, and secure data storage systems is essential to support widespread adoption of digital pathology.
2) Capacity Building: Structured training programs and continuous professional development initiatives should be implemented to enhance pathologists’ competence and confidence in digital diagnostics.
3) Quality Assurance and Standardization: Development of national guidelines and standard operating procedures for digital pathology will be critical to ensure consistency, accuracy, and regulatory compliance.
4) Integration with National Health Systems: BHIMS and similar platforms should be integrated with existing health information systems to enable seamless data sharing, reporting, and surveillance.
5) Adoption of Advanced Technologies: Incorporating AI and computer-assisted diagnostic tools may improve detection of subtle histopathological features and reduce observer variability.
6) Policy and Financing Frameworks: Government commitment and stakeholder engagement will be essential to establish supportive policies, mobilize resources, and ensure long-term sustainability of digital pathology initiatives.

This study provides compelling evidence that digital histopathology is a reliable and scalable alternative to conventional microscopy in low-resource settings. While some limitations remain in the assessment of nuanced features, these can be addressed through targeted investments in technology, training, and quality assurance. The successful implementation of BHIMS highlights the transformative potential of digital health innovations to expand access to pathology services, strengthen cancer care systems, and ultimately improve patient outcomes in Uganda and similar contexts.

### Limitations and future work

Despite its promise, the digitization process was constrained by several technical limitations. Static image scanning, rather than whole-slide imaging, was used due to equipment constraints. This limited the ability to navigate tissue sections at varying depths or resolutions, possibly contributing to lower agreement in complex features such as mitoses and invasion Jahn et al. (2020) emphasized that such limitations call for investments in whole-slide scanners, stable internet connectivity, and robust digital storage infrastructure.

Furthermore, the study underscores the need for targeted capacity building. Focused training in digital morphology interpretation, particularly for subtle features—could enhance diagnostic consistency. The integration of AI tools to assist in the detection of mitotic figures or lymphovascular invasion may also improve diagnostic precision (Jahn et al. 2020; Azam et al. 2021).

This study did not include a formal qualitative assessment of user perspectives. Future research should incorporate qualitative methods to better understand user experiences and workflow challenges, thereby informing improvements in system usability and adoption. Additionally, the study lacked a comprehensive economic evaluation and detailed policy analysis, limiting the ability to draw conclusions regarding scalability and long-term sustainability.

## Conclusion

These findings affirm the reliability of digital pathology for consistent intra-observer diagnostic interpretations, especially for core diagnostic features such as cancer type and diagnosis. Digital pathology, supported by systems like BHIMS, offers a scalable and effective model for enhancing diagnostic reach and quality in low-resource settings. However, to fully realize its potential, investments in technology, training, and supportive infrastructure are essential—particularly for improving consistency in evaluating nuanced histological characteristics.

## Data Availability

The datasets used and/or analyzed during the current study are available from the corresponding author on reasonable request.
